# Enhanced Gray Matter Volume Compensates for Decreased Brain Activity in the Ocular Motor Area in Children with Anisometropic Amblyopia

**DOI:** 10.1155/2020/8060869

**Published:** 2020-04-16

**Authors:** Weizhao Lu, Xueliang Yu, Lisheng Zhao, Yanli Zhang, Feng Zhao, Yi Wang, Jianfeng Qiu

**Affiliations:** ^1^Medical Engineering and Technology Research Center, Shandong First Medical University & Shandong Academy of Medical Sciences, Tai'an, Shandong, China; ^2^Department of Ophthalmology, Dezhou Municipal Hospital, Dezhou, Shandong, China; ^3^Department of Radiology, Dezhou Municipal Hospital, Dezhou, Shandong, China; ^4^Department of Ophthalmology, The Second Affiliated Hospital of Shandong First Medical University, Tai'an, Shandong, China

## Abstract

**Purpose:**

Anisometropic amblyopia usually occurs during early childhood and results in monocular visual deficit. Recent neuroimaging studies have demonstrated structural and functional alterations in pediatric anisometropic amblyopia (PAA) patients. However, the relationship between structural and functional alterations remains largely unknown. The aim of this study was to investigate the relationship between structural and functional alterations in PAA patients.

**Materials and Methods:**

Eighteen PAA patients and 14 healthy children underwent a multimodal MRI scanning including T1WI and functional MRI (fMRI). Voxel-based morphometry was used to assess structural alterations between PAA patients and healthy children. Regional homogeneity (ReHo) was used to investigate changes in local spontaneous brain activity in the enrolled subjects. Correlations between structural, functional alterations, and clinical information were analyzed in the PAA group.

**Results:**

Compared with healthy children, PAA patients exhibited significantly reduced ReHo of spontaneous brain activity in the right superior temporal gyrus (STG) and right middle frontal gyrus (MFG) and increased gray matter volume in the right lobules 4 and 5 of the cerebellum. The gray matter volume of the right lobules 4 and 5 of the cerebellum was negatively correlated with the ReHo values of the right MFG.

**Conclusions:**

Our findings may suggest that PAA patients experience structural and functional abnormalities in brain regions related to oculomotor and visual-spatial information. In addition, the increased gray matter volume may compensate the decreased brain activity in the oculomotor regions, which reflects compensatory or neural plasticity in PAA patients.

## 1. Introduction

Amblyopia is a disorder of the visual system in the absence of any detectable pathologic or structural abnormalities of the eye [1, 2]. It is a common cause of monocular visual deficit in children and affects approximately 1% to 5% of the children worldwide [3, 4]. Amblyopia is clinically divided into different types according to the eye abnormalities responsible for disrupting visual development [5]. Among different categories, anisometropic amblyopia is a common type characterized by a difference greater than 2.5 diopters in refractive power between 2 fellow eyes [5, 6]. Anisometropic amblyopia is of great clinical interest as a well-known identifiable amblyogenic factor in children [3, 7]. The amblyogenic factors interfere with normal development of the visual pathways during maturation [1, 8]. The result is structural and functional impairment of the visual cortex and central nervous system [8].

Although previously viewed as a disorder in the visual system, anisometropic amblyopia is associated with more neurological abnormalities in the central nervous system [5], and the neural mechanisms of anisometropic amblyopia are still not entirely clear [1]. Recent advances in neuroimaging techniques have enabled the exploration of brain regions associated with pediatric anisometropic amblyopia (PAA) patients. The blood oxygen level-dependent imaging technique of resting-state functional magnetic resonance imaging (rs-fMRI) has been widely used to explore brain functional changes in amblyopia patients [9–12]. rs-fMRI studies have demonstrated altered brain activity both in the visual cortices and in the postcentral gyrus and precuneus gyrus both in amblyopic children and adults [2, 9]. The rs-fMRI functional connectivity study has revealed functional connectivity changes between the visual area and the cerebellum and inferior parietal lobule [10]. The task fMRI has provided evidence that there were abnormalities in the cortical processing of motion in amblyopia patients [11–13].

Structural MRI, as one of the routine clinical MRI techniques, allows direct access to the anatomy of the brain [6]. Morphometric studies in children with amblyopia have consistently demonstrated alterations of gray matter regions involved in visual processing [6]. Voxel-based analysis has detected gray matter volume alterations in the primary visual cortex, middle frontal gyrus, inferior temporal gyrus, and other regions related to visual function in children with amblyopia [14–16]. Surface-based morphometry study has demonstrated decreased cortical thickness in the primary visual cortex and higher visual cortex of children with amblyopia and altered mean curvature of the cerebral cortex [5].

Although many neuroimage studies have found abnormal brain activity and brain morphometry in children with amblyopia, few studies have focused on multimodal techniques. Single-modal MRI studies only demonstrated brain alterations in morphometry, structure, or function and had low reproducibility [17]. Multimodal MRI studies could not only demonstrate brain alterations from different prospect but also reveal joint information from different MRI modalities [18]. Therefore, it is reasonable to expect that if children with anisometropic amblyopia are associated with changes in both structural and functional measures, then it is hypothesized that a small number of joint information would best capture the differences between the patients and controls. In this study, regional homogeneity (ReHo) was applied as the functional measure and the gray matter volume was used as the structural MRI measure, and it was expected to find joint information which would suggest neural covariation or neural compensatory mechanism.

## 2. Materials and Methods

### 2.1. Participants

This cross-sectional study was approved by the Institutional Review Board of Dezhou Municipal Hospital and Shandong First Medical University in accordance with the Declaration of Helsinki. Written informed consent was obtained from all the subjects or their legal guardians.

The enrollment criteria for PAA patients were (1) 6-12 years old, (2) right-handed, (3) diagnosed with anisometropic amblyopia characterized by visual acuity of amblyopic eyes ≤0.8 (decimal representation), visual acuity of fellow eyes ≥0.8, and anisometropia ≥2.5 diopter of spherical equivalent, and (4) those with amblyopia in the left eyes. In this study, patients were excluded if they had (1) ocular cause for reduced acuity, (2) prior spectacle wear or other treatment for amblyopia, (3) myopia more than a spherical equivalent of -6.00 diopter in the amblyopic eye, (4) history of psychiatric or neurological illnesses, and (5) MRI contraindications such as implanted sternal devices and heart pacemaker. For healthy children, the enrollment criteria were (1) 6-12 years old, (2) right-handed, (3) those with no ocular pathology or abnormal visual development, (4) those with no history of psychiatric or neurological illnesses, and (5) those with no MRI contraindications.

In total, 18 PAA patients and 14 healthy children were enrolled in the study. Two patients had excessive head motion during fMRI scanning and were excluded, leaving 16 PAA patients and 14 healthy children in the statistical analysis. All participants received detailed eye examinations that included assessments of visual acuity and optical coherence tomography.

### 2.2. Multimodal MRI Examination

All enrolled children underwent multimodal MRI scanning using a 1.5 Tesla MR scanner (GE Optima MR360). Structural MRI images were firstly acquired using a spin echo sequence in a sagittal orientation. The scan parameters were as follows: repetition time = 1750 ms, echo time = 20.43 ms, inversion time = 720 ms, number of signal averages = 1, flip angle = 90°, field of view = 240 mm × 240 mm, matrix = 256 × 256, slice thickness = 1 mm, and 176 sagittal slices with no gap covering the whole brain.

A resting-state fMRI scan were then conducted using an echo planar imaging sequence with the following parameters: repetition time = 3000 ms, echo time = 40 ms, field of view = 240 mm × 240 mm, matrix = 64 × 64, slice number = 31, slice thickness = 5 mm, slice gap = 0 mm, flip angle = 90°, and scan duration time = 384 s (128 volumes).

### 2.3. Multimodal MRI Data Processing

For each child, fMRI data preprocessing followed the common steps [19]: (1) DICOM to NIFTI conversion; (2) the first 10 volumes of functional time series were discarded for fMRI signal to reach a steady-state magnetization; (3) slice timing; (4) head motion correction (to eliminate the effect of head motion, exclusion thresholds were set at maximum translation >2 mm or maximum head rotation >2 degree; two PAA patients was excluded); (5) fMRI data were then normalized to the Chinese pediatric templates using a two-step normalization process with the help of structural images [20]; (6) linear detrend of functional time courses; (7) six head motion parameters, white matter, and cerebrospinal fluid signal were considered as nuisance covariates and regressed out; and (8) a band-pass filter (0.01-0.08 Hz) was performed on the fMRI time series.

ReHo calculation was performed on the preprocessed fMRI data to generate the ReHo map. In the ReHo map, the value of each voxel equals to the Kendall's coefficient concordance (KCC) of the voxel with its adjacent 27 voxels [19, 21]. Then the ReHo map was smoothed using a Gaussian kernel of 6 mm FWHM.

Structural image preprocessing steps were as follows: (1) The structural images were spatially registered to the Chinese pediatric templates by a nonlinear registration. (2) The images were then segmented into gray matter, white matter, and cerebrospinal fluid according to the templates using the segment module of SPM8. (3) Gray matter images were smoothed with an isotropic Gaussian kernel of 6 mm FWHM.

### 2.4. Statistical Analysis

Statistical analysis of demographic and clinical information between PAA group and healthy children were performed using SPSS 20.0. Age, visus oculi dextri (VOD), visus oculi sinistri (VOS), and an amount of anisometropia were assessed by independent *t*-test and gender was assessed by chi-squared test. *p* value < 0.001 was considered statistically significant.

The general linear model was applied for statistical analysis of multimodal MRI measures (ReHo and gray matter volume) between PAA and healthy children with age and gender considered as nuisance covariates. Statistical difference was set as *p* < 0.001. The value of Cohen *d* was used to describe the effect size (ES) of significant clusters.

### 2.5. Correlation Analysis

First, we explored the correlation between mean the ReHo values, mean gray matter volume of the functional, structural alterations, and clinical information in the patient group via partial correlation analysis to factor out age and gender. Second, we assessed the relationship between the ReHo values of functional alterations and gray matter volume of the structural alterations using Pearson's correlation analysis. *p* < 0.05 was considered to be statistically significant.

## 3. Results

### 3.1. Demographic and Clinical Information

Table 1 lists the demographic and clinical information of the PAA group and healthy children. The two groups were well matched in terms of age (*p* = 0.182) and gender (*p* = 0.699). However, there were significant differences in VOD (*p* = 0.012), VOS (*p* < 0.001), and amount of anisometropia (*p* < 0.001) between the two groups.

### 3.2. ReHo and VBM Analysis

**Figure 1:**
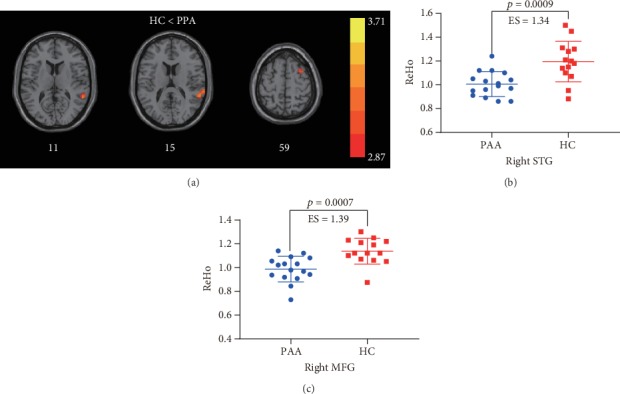
Comparisons in ReHo values between healthy controls and PAA patients (*p* < 0.001). (a) Comparisons of the whole-brain ReHo maps. (b) Comparison of ReHo values in the right STG. (c) Comparison of ReHo values in the right MFG. ES: effect size. The error bar represents mean ± standard deviation.

Compared with healthy children, PAA patients showed lower ReHo values in the right superior temporal gyrus (STG) and right middle frontal gyrus (MFG) (*p* < 0.001) as shown in Figure 1 and Table 2.

**Figure 2:**
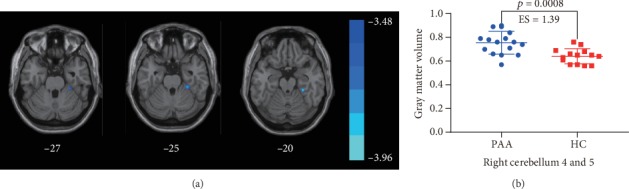
Comparisons in gray matter volume between healthy controls and PAA patients (*p* < 0.001). (a) Comparisons of the whole-brain gray matter volume. (b) Comparison of the gray matter volume in the right lobules 4 and 5 of the cerebellum. ES: effect size. The error bar represents mean ± standard variation.

VBM demonstrated an increased gray matter volume in the right lobules 4 and 5 of the cerebellum (cerebellum 4 and 5) and the right fusiform gyrus (FG) in the PAA brain compared with healthy children. The VBM analysis result is illustrated in Figure 2. The brain regions with significant VBM differences are identified in Table 3.

### 3.3. Correlation Analysis

**Figure 3:**
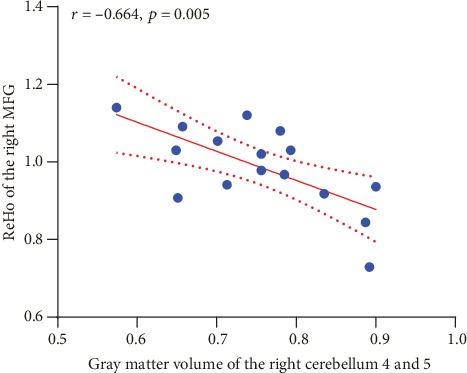
Correlation between the gray matter volume in the right lobules 4 and 5 of the cerebellum and ReHo values in the right MFG. The area between the dashed lines is the 95% confidence interval.

There were no significant correlations between clinical information and multimodal MRI measures in the PAA group. However, the ReHo values of the right MFG were negatively correlated with the gray matter volume of the right lobules 4 and 5 of the cerebellum (*p* = 0.005, *r* = −0.664), as illustrated in Figure 3.

## 4. Discussion

In a previous study, we analyzed functional alterations in high-tension glaucoma patients and found changes in ReHo values in the high-tension glaucoma brain [19]. In the current study, we applied the same functional measures to children with anisometropic amblyopia. In addition, we also analyzed structural alterations in children with anisometropic amblyopia using gray matter volume as structural measure.

Amblyopia was present in the left eyes for the enrolled PAA patients in this study. It is known that visual information from the left eyes go to the right hemisphere of brain and vice versa. The brain regions with decreased ReHo values and increased gray matter volume were all in the right hemisphere (amblyopic eye side), which indicated amblyopia-related brain alterations. In addition, we found negative correlations between the gray matter volume of the right lobules 4 and 5 of the cerebellum and ReHo values of the right MFG. The result would indicate that the decreased spontaneous activity of anisometropic amblyopia is secondary to the visual impairment and the increased gray matter volume may play a beneficial role in children with anisometropic amblyopia.

In children with anisometropic amblyopia, we found lower ReHo values in the right MFG (amblyopic eye side). The decreased ReHo values have been considered to reflect decreased brain spontaneous activity [21]. The MFG is part of frontal eye field (FEF) which is responsible for saccadic eye movement and voluntary eye movement [22]. For PAA patients in the present study, visual impairment and abnormal binocular vision disturb the normal development of visual function in the FEF, which may result in decreased spontaneous activity in the FEF. Several state-of-the-art studies have demonstrated patients with anisometropic amblyopia have prolonged saccade latency and decreased precision [23, 24]; the decreased brain spontaneous activity may give potential explanation to the abnormalities in saccadic eye movements. Furthermore, the MFG is thought to contribute to working memory, inhibition, and higher cognitive functions [25]. Previous fMRI studies have revealed decreased brain spontaneous activity of the MFG in patients with amblyopia [2, 26]. In line with the previous findings [2, 26], the decreased ReHo indices may reflect decreased integration abilities in anisometropic amblyopia.

The present study also revealed decreased brain spontaneous activity in the right STG (amblyopic eye side) in PAA patients. The STG, alongside with the middle temporal visual area (V5/MT+), is an important part of the dorsal visual pathway [27]. The dorsal visual pathway is involved in visual-spatial information such as motion sensation and space recognition [28]. The previous fMRI study in patients with amblyopia attributed abnormalities in this area to impaired function of visual-spatial functions [29]. In addition, the STG is the site of auditory cortex responsible for auditory information processing [30]. Decreased brain activity in this region may reflect impaired visual-auditory integration in PAA patients.

In PAA patients, increased gray matter volumes in the right lobules 4 and 5 of the cerebellum and the right FG (amblyopic eye side) were found. Furthermore, the gray matter volume of the right lobules 4 and 5 of the cerebellum was negatively correlated with brain spontaneous activity in the right MFG. The FG is related to higher visual functions such as face recognition [31]. In PAA patients, visual deficit and decreased contrast sensitivity could add visual noise in face processing, which might lead to noise-induced neuron activities in the FG and eventually lead to increased gray matter volume in the FG [31].

As discussed above, the MFG was related to oculomotor pathway of saccadic eye movement [32]. The cerebellum, which functionally interacts with the FEF, is also involved in the control of eye movements just like the MFG [2, 33]. There is a close relationship between structure and function in the human brain [34]. The integrity of any particular region could be important bilaterally, and in fact, one hemisphere may “compensate” for the damage in the opposite hemisphere [34].

As is known, there is a specific three-level hierarchy in the oculomotor system responsible for saccadic eye movements [35]. The lowest level is in charge of saccade execution. The cerebellum is in the second level of the oculomotor system responsible for target selection. At the highest level are the FEF, parietal eye field, and the supplementary eye field [35]. In anisometropic amblyopia, positional uncertainty and eccentricity of fixation are common visual deficits [36]; these visual deficits may lead to decreased spontaneous activity in the FEF. However, the need for eye realignment to reduce the consequences led by these visual deficits may have contributed to a compensatory increase of gray matter volume and plasticity in the oculomotor processing regions in the second and third levels. So the decreased ReHo values in the right MFG could be compensated by the increased gray matter volume in the right cerebellum, which may reflect neural plasticity [32, 37]. Logically, increased gray matter volume may benefit PAA patients with visual deficits. However, whether PAA patients may benefit from the increased gray matter volume is not clear. Future studies are needed to address this issue.

Previous studies on amblyopia have shown reduction of brain spontaneous activity and gray matter volume in the visual cortices [9, 14, 15], which are inconsistent with our findings. This discrepancy may be explained by the following reasons. First, the present study sample was a narrow range of anisometropic amblyopia children, whereas the previous studies enrolled a broad type of amblyopia patients (strabismic amblyopia, anisometropic amblyopia) with both children and adults [9, 14, 15]. Different types of amblyopia have different pathological mechanisms and temporospatial characteristics, which may lead to differences in the neurophysiological mechanisms between different types of amblyopia [38]. Differences in age also lead to differences in the MRI measures [39]. Second, a previous study has demonstrated neural compensatory plasticity inside the visual cortices [2]. Neural plasticity may occur in the visual cortices in the present study cohort.

## 5. Limitations

In this study, we did not collect enough clinical data to investigate the links between amblyopic information and MRI measures such as eye movements. In addition, most of statistical analyses reported in this study did not reach statistical significance when corrected for multiple comparisons. This may be due to a small sample size. Further studies with larger sample size and more clinical data are needed to validate our present findings.

## 6. Conclusion

In conclusion, this study applied structural and functional MRI to study brain changes in PAA patients. The results demonstrated that there were structural and functional alterations in the oculomotor processing regions, dorsal visual pathway, and higher visual areas in PAA patients. The enhanced gray matter volume in the oculomotor regions may reflect a compensatory mechanism for visual deficits resulting from functional impairment of the FEF. Future studies with larger sample size and saccade task experiment are needed to consolidate the present findings and explore the oculomotor regions in PAA patients.

## Figures and Tables

**Table 1:** Demographic and clinical information of pediatric anisometropic amblyopia patients and healthy children.

	PAA	HC	*p* value
Age (years old)	6.93 ± 2.28^a^	8.14 ± 2.23^b^	0.182^c^
Sex (M/F)	10/6	8/6	0.699^d^
VOD (decimal representation)	0.879 ± 0.1611	1	0.012^c^
VOS (decimal representation)	0.3571 ± 0.1842	1	<0.001^c^
Amount of anisometropia (diopter)	3.4464 ± 1.0781	0	<0.001^c^

HC: healthy children. ^a^The range and median of age in PAA group were [6, 12] and 7. ^b^The range and median of age in control group were also [6, 12] and 7. ^c^The *p* value was calculated using the independent *t*-test. ^d^The *p* value was calculated using the chi-squared test.

**Table 2:** Brain regions with ReHo differences between PAA patients and healthy controls (*p* < 0.001).

Conditions	Brain region	BA	Cluster volume (mm^3^)	MNI coordinates			*T* value
				*X*	*Y*	*Z*	
HC > PAA	Right STG	22, 42	837	57	-42	15	3.4234
HC > PAA	Right MFG	6	405	30	9	60	3.1717

HC: healthy children; BA: Brodmann area; MNI: Montreal Neurological Institute.

**Table 3:** Brain regions with gray matter volume differences between pediatric anisometropic amblyopia patients and healthy controls (*p* < 0.001).

Conditions	Brain region	BA	Cluster volume (mm^3^)	MNI coordinates			*T* value
				*X*	*Y*	*Z*	
HC < PAA	Right cerebellum 4 and 5 Right FG	20, 35, 36	504	30	9	60	-3.9619

HC: healthy children; BA: Brodmann area; MNI: Montreal Neurological Institute.

## Data Availability

The MRI data used to support the findings of this study are available from the corresponding author upon request.
